# Allergens in Allergy Diagnosis: A Glimpse at Emerging New Concepts and Methodologies

**Published:** 2012-10-11

**Authors:** Ivana Giangrieco, Chiara Rafaiani, Marina Liso, Paola Palazzo, Debora Pomponi, Lisa Tuppo, Roberta Crescenzo, Maurizio Tamburrini, Adriano Mari, Maria Antonietta Ciardiello

**Affiliations:** 1 Institute of Protein Biochemistry, CNR, Naples, Italy; 2 Center for Molecular Allergology, IDI-IRCCS, Rome, Italy

**Keywords:** Food allergens, ISAC microarray, test systems

## Abstract

Allergic diseases are important concern of public health. A reliable diagnosis is of utmost importance for the management of allergic patients both when immunotherapy is planned and when the treatment is essentially based on the avoidance of the allergy source. However, the available diagnostic systems sometimes fail to detect specific IgE antibodies thus impairing the correct diagnosis. The traditional test systems are generally based on the use of protein extracts derived from the allergenic sources whose composition is very variable and cannot be standardized. The development of a new methodology combining the so-called allergenic molecule-based diagnosis with the multiplex microarray technology and allowing the analysis of multiple purified allergens in a single test represents an important improvement in allergy diagnosis. In addition, the biochemical and immunological characterisation of individual allergens has provided new insights into the understanding of allergen-IgE recognition that could be exploited for further improvements of allergy diagnostic tests.

## INTRODUCTION

The prevalence of allergic diseases, affecting both children and adults, is reported to be on the rise and these pathologies are no longer confined to specific seasons, or to people living in specific areas [1–5]. It has been estimated that 25% of the population worldwide suffer from this problem, and within this number 1–2% of adults and 5–7% of children suffer from food allergies [6]. The causes of the epidemic spread of reported allergies are still unclear and may be due to a combination of different factors. Various hypotheses have been made associating such an increase to an increased hygiene levels (the so-called “hygiene hypothesis’), to a genetic proneness, to the increasing use of products deriving from industrial food-processing and to other unknown reasons [5,7–10].

The effect of globalization is also included in the list of causes that seem to play a key role in the spread of allergic reactions. In fact, not only populations migrate but also foods. A classic example is the allergy to kiwifruit. Some decades ago, this allergy was absent in geographical areas such as Europe and USA because no one ever ate kiwifruit at that time. Now many people eat this fruit and lots of kiwifruit allergies have been described [11]. These aspects may strongly affect the widespread proliferation of food allergies.

It is actually very difficult to have exact epidemiological data on this topic. In addition, a discrepancy between the rates of perceived food allergy and the rates of true allergy has been observed. In fact, literature reports state that people have a perception of food allergy even four times higher than that confirmed by available allergy tests [11]. A possible explanation is that people sometimes confuse allergy with intolerance (due to metabolic conditions, such as lactose intolerance, or other immunological mechanisms as in coeliac disease) or with mild food poisoning [12–13]. Some discrepancies could also be due to some false results provided by the available diagnostic systems. In fact, depending on the methodology and reagents used, *in vitro* serological tests (RAST, ImmunoCAP) and *in vivo* tests (skin prick test (SPT), prick-by-prick test) sometimes can provide false negative responses [14]. In some cases, it seems that the only way to assess the allergy to a food is by feeding/challenging the patient with the suspected food. However, this practice is risky mostly when applied to subjects reporting severe allergic reactions. Therefore, to obtain a reliable and safe diagnosis it seems mandatory to improve *in vitro* or *ex vivo* allergy diagnostic systems [15].

## THE ALLERGIC REACTION

**Fig. 1:**
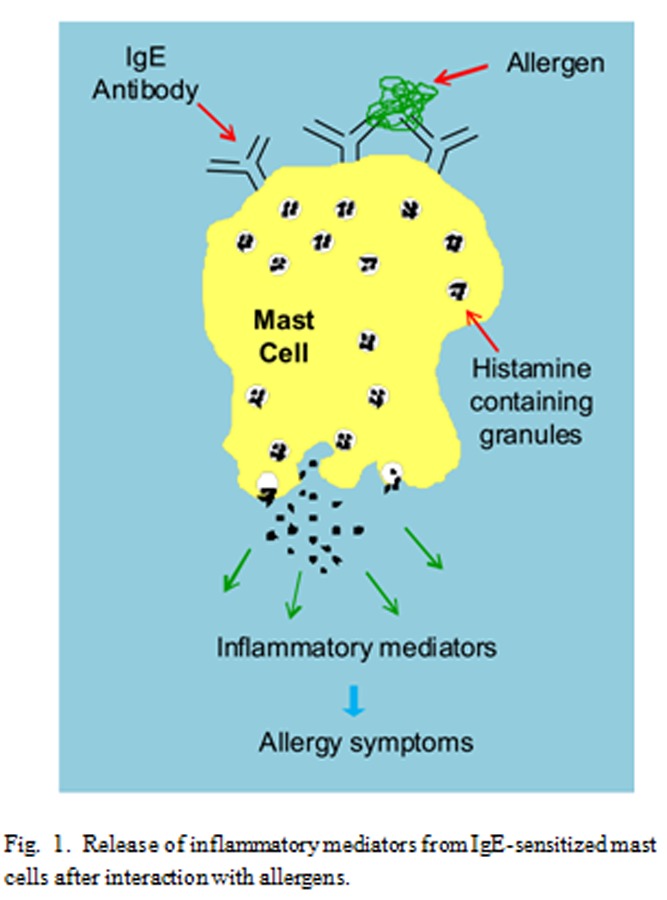
Release of inflammatory mediators from IgE-sensitized mast cells after interaction with allergens.

An allergic reaction is an abnormal response of the human body when in contact with an allergen. The most common allergen sources of protein nature include house dust mite, pollens from grasses, weeds and trees, animal dander (including cat, dog and horse), moulds, foods (including tree nuts, peanuts, shellfish, fish, milk, eggs, wheat, fruits), hymenoptera venoms, and latex. When a sensitive subject is exposed to the allergy source a type of white blood cells (B lymphocytes) produce a specific antibody known as Immunoglobulin E (IgE) against the allergenic molecule(s) contained in that stuff (primary response or sensitization). The IgE then binds to another type of white blood cell (mast cells) by mean of a specific high affinity receptor (FCεR), and when the mast cells come into contact with that allergen(s) again, they initiate a complex immune response, involving the release of preformed or neo-formed inflammation mediators, that cause the allergy symptoms (Figure 1). The allergic reaction may cause one or more symptoms that may be more or less severe, including urticaria, rhinitis, conjunctivitis, angioedema, oral allergic syndrome, abdominal pain, diarrhea, asthma, anaphylactic shock. Mainly for those people showing severe symptoms, it is important to be able to correctly identify the allergenic sources to which the patient reacts.

## DIAGNOSIS: ALLERGIC, OR NOT ALLERGIC, THIS IS THE QUESTION

The traditional testing systems are generally based on the use of commercially available protein extracts derived from the allergenic sources. However, they frequently fail to detect specific IgE because their composition can be very variable. Ripening stage, post-harvest treatments, differences among cultivars, proteolytic degradation and protocols used for the extraction significantly affect the relative amounts of many proteins and the profile of allergenic components [16–18].

Since it seems impossible to obtain standardized extracts with a constant allergenic composition and containing all the allergenic proteins present in the natural source, molecule-based diagnosis has gained more attention in the recent past. In fact, the scientific research and the companies are evolving through the development of new technologies useful for the detection of specific IgE against individual allergenic molecules. Unlike traditional systems, these won’t use protein extracts, but only natural or recombinant purified allergens, that in this way will allow a better standardization of the whole system.

### The multiplex microarray-based technology of the “ISAC system”: a new methodology

**Fig. 2:**
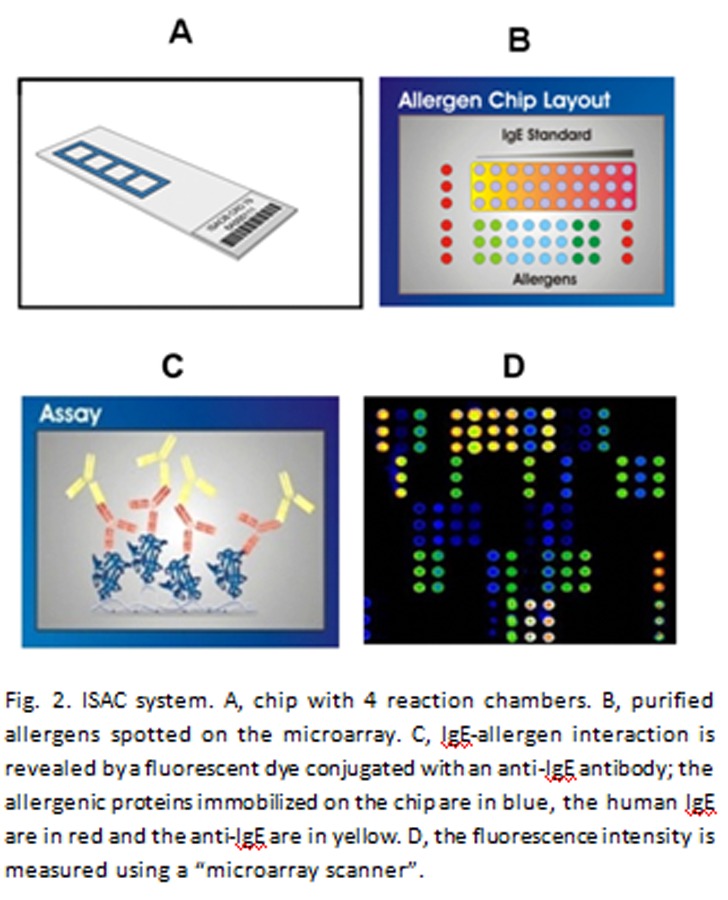
ISAC system. A, chip with 4 reaction chambers. B, purified allergens spotted on the microarray. C, IgE-allergen interaction is revealed by a fluorescent dye conjugated with an anti-IgE antibody; the allergenic proteins immobilized on the chip are in blue, the human IgE are in red and the anti-IgE are in yellow. D, the fluorescence intensity is measured using a “microarray scanner”.

ISAC (Immuno Solid-phase Allergen Chip) is an *in vitro* diagnostic system useful for semiquantitative analysis of IgE in serum samples [19–22]. In contrast with the traditional systems, it uses only purified allergens. It is made of a microscope glass slide containing four identical reaction chambers (Figure 2A) and each chamber is a microarray where individual allergens are immobilized separately (Figure 2B). The IgE contained in the serum of an allergic subject recognize one or more immobilized allergens on the microarray (Figure 2C) and the interaction is revealed using a secondary antibody labeled with a fluorescent probe, specific for human IgE (Figure 2D).

This multiplex microarray-based technology allows the simultaneous measurement of IgE antibodies specific for different individual allergens (i.e. multiplex analysis) with the same serum sample [21–23]. Therefore it can be exploited to perform some steps of the classic procedures easier and faster. In some allergy centers, it is routinely used for allergy diagnosis providing information on the subjects’ sensitisation to all the allergens available on the microarray with a single test [21–22,24–25].

Few years ago the first version of the ISAC microarray became commercially available. It had 74 different allergenic proteins spotted on the microarray (ISAC74). The number of allergens immobilized on this microarray is growing and at present a version with 112 allergens (ISAC112) is available.

There are other diagnostic systems (i.e. ImmunoCAP, Immulite) that recently started to use allergenic molecules, that is the purified allergens rather than the raw extracts, for allergy diagnosis. However, they can analyze only one allergen for each test (IgE singleplex testing).

The ISAC system, providing IgE multiplex testing, adds important advantages to the singleplex testing. For instance, the ISAC system is a time-saving and money-saving methodology because information about many allergens is obtained with a single test. An additional advantage is the possibility to detect the IgE reactivity towards hidden or unsuspected allergens/allergy sources. In fact, sometimes the allergic reaction can be caused by a contamination of foods that can contain unexpected components. For instance, if a subject reports the appearance of allergic symptoms after the ingestion of chicken and after the ingestion of fish, the allergologist that uses the singleplex test systems will test the reactivity to chicken and to fish.

**Fig. 3:**
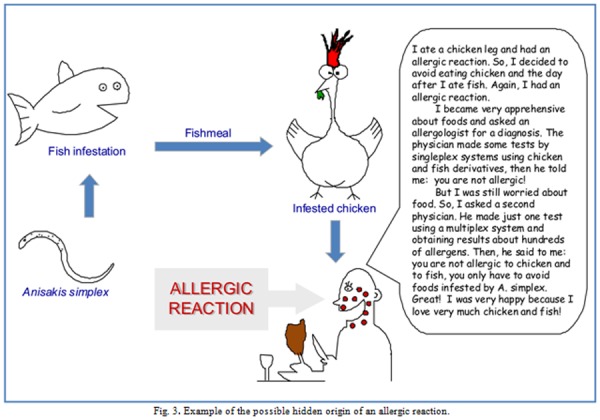
Example of the possible hidden origin of an allergic reaction.

If the response of the testing is negative, then the allergy trigger will remain a mistery and the subject will exclude chicken and fish from his/her diet (Figure 3). In contrast, if the allergologist uses a multiplex system the response could reveal the presence in the subject’s serum of IgE specific for components of unsuspected allergy sources, such as spices or, for example, for the fish parasite *Anisakis simplex*, whose allergenic components have been detected even in chicken fed with seafood infested by it [26]. Therefore, the multiplex system can reveal sensitisation sources even when the patient does not or cannot report indications about the allergy triggers [21].

### Does one allergen fit all the homologs? …be careful!

Some allergens are widely distributed in taxonomically different organisms [27]. For instance, important plant food allergens, such as the Lipid Transfer Proteins (LTP), are quite ubiquitous proteins. It means that homologs of LTP can be present in many, if not all, botanically different plant-derived foods.

**Fig. 4:**
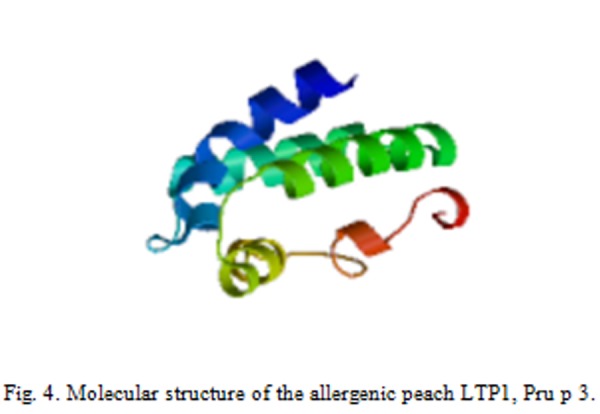
Molecular structure of the allergenic peach LTP1, Pru p 3.

Up to now LTP from 63 different plant sources have been reported as allergens (www.allergome.org) [28], 46 of them are present in edible parts of plants and all of them belong to the LTP1 protein subfamily. Among allergenic LTPs, the best characterized at the structural, immunological and clinical level is the peach LTP, Pru p 3 (Figure 4).

LTP is a small allergen (9 kDa) that can cause severe symptoms, including the anaphylactic shock, and one of the most important sensitizer in the Mediterranean area [29–32]. In allergy diagnosis, there is the trend to use the peach LTP, Pru p 3, to assess allergy to all the plant LTP. This could be a reasonable procedure if all homologous LTPs had identical epitopes recognized by IgE. Unfortunately, this seems not to be the case, otherwise a subject allergic to LTP should react and avoid any plant-derived food. Several reviews on the topic of LTP reported preliminary evidence of a heterogeneous immunological behaviour of this group of molecules [33–34]. A recent paper reported a comparative study of peach and kiwifruit LTPs showing by *in vitro* and *in vivo* tests that some subjects were IgE positive to some LTPs and negative to some others [35]. In this study, in addition to subjects showing positive reaction to both peach and kiwifruit LTP, few subjects reacted only to LTP from one fruit and did not react to the other. The results obtained by *in vivo* tests on allergic subjects could be ascribed to the heterogeneous epitope pattern present in the different LTPs. These results underline that the concept “one allergen fits all the homologs”, that is sometimes applied [36–38], may produce some erroneous diagnosis. The comparative study of kiwifruit and peach LTPs [35] clearly indicates that the biochemical grouping of allergens can be misleading in the allergy diagnosis and that an improvement can be obtained by testing every single patient with the most comprehensive panel of available LTPs/allergens. The observation that some subjects have isolated IgE positivity to single LTP/allergens provides useful information on what to exclude but, most importantly, on what to leave in patient’s diet.

### Allergens are proteins…don’t forget it!

Allergens have a protein nature, therefore they have all the properties of protein molecules. It is well-known that the protein structure and function is strongly affected by the chemical and physical features of the environment, including the chemical composition of the medium, ionic strenght, pH, temperature, etc. The function of some proteins is implemented by the interaction with another protein molecule. This protein-protein interaction can occurr or not, it depends on the environmental/experimental conditions around the two actors.

Allergy diagnosis is based on the detection of a protein-protein interaction, involving the allergen and the IgE antibody specifically recognizing the allergen under investigation. The available diagnostic systems apply the same experimental condition (phosphate buffered saline at neutral pH) to evaluate the sensitivity to any allergen, independently of the characteristics of the allergy source or the environment that an allergen can encounter during, for example, the transit through the gastrointestinal tract.

Recently, a study focused on kiwellin (allergen Act d 5 [39]) has shown that the number of subjects showing a positive *in vivo* test (SPT) to this kiwifruit allergen was increased when, in addition to the standard protocol, conditions more similar to those present in kiwifruit were used to solubilize the allergen [40]. The observation that some subjects had a positive reaction either at neutral or acidic pH values suggests that this allergen, depending on the environmental conditions, may expose different epitopes. Nevertheless, some subjects may have IgE antibodies recognizing epitopes exposed in each of the two investigated environmental conditions. The conformational analysis by circular dichroism measurements in different experimental conditions indicate that Act d 5 3D structure is modulated by the solvent pH and polarity [40]. Therefore, Act d 5 displays pH-driven conformational changes, but this effect is more evident in a low-dielectric costant medium, that is representative of several cellular environments [41], like for instance the interior of biological membranes. During the transit in the gastrointestinal system, Act d 5 can encounter environments characterized by different pH values, ranging from the very acidic one of the stomach to values close to neutrality, and can meet the hydrophobic environments of the cell membranes. Therefore, it may be hypothesized that, depending on the environments encountered, this allergen may undergo *in vivo* conformational changes and expose different epitopes, inducing the synthesis/interaction of different specific IgEs.

## CONCLUDING REMARKS

Although in some cases the immunotherapy can be suggested, the best treatment of allergic subjects is still based on the avoidance of the allergen source. In the case of a food allergy, it means the exclusion of specific foods from the diet. The implication is that a proper management of these subjects requires a highly reliable diagnosis in order to identify the allergenic molecules/sources that each patient has to avoid. However, the widespread diagnostic systems, based on the use of raw protein extracts, do not provide reliable responses. Nevertheless, recent studies indicate that allergy diagnosis can be improved by dealing with different aspects ranging from the choice of new methodologies to the accurate selection of reagents and of experimental conditions to be applied. So, in perspective, we could reach a more reliable allergy diagnosis if some new concepts and methodologies will be welcomed and applied.

For instance, the new systems based on allergenic molecules will provide an increased reliability of the allergy testing results because the use of purified allergens allows a better standardization of the diagnostic tests. The multiplex biochip-based immunoassay (ISAC system) represents an additional improvement because it makes possible the analysis of the reactivity towards more than one hundred allergens with a single test, using only 20 μl of serum. Furthermore, it allows the detection of unsuspected sensitivities caused by hidden allergens because no preselection of allergenic molecules to be tested is applied.

Recent literature reports suggest that the inclusion in the diagnostic systems of a panel of homologous components from different sources, rather than one component chosen to represent all the homologs, will also contribute to the improvement in the accuracy of allergy diagnosis. In fact, the recent literature shows that homologous proteins, grouped in a single family on the basis of their biochemical features, can have different immunological properties, that means that “one allergen does not fit its homologs”.

Additional improvements of both *in vivo* and *in vitro* allergy tests can be reached by exploiting the observation that the physico-chemical features of the experimental conditions used to make the test can affect the allergen properties, thus affecting the allergen-IgE interaction and therefore the response of the diagnostic test.
